# Analysis of vertical AND flash memory for energy-efficient, scalable, fast CIM beyond vertical NAND flash memory

**DOI:** 10.1186/s40580-026-00547-z

**Published:** 2026-04-24

**Authors:** Jonghyun Ko, Jiseong Im, Sung-Ho Park, Jahyun Gu, Joonhyung Cho, Joon Hwang, Ryun-Han Koo, Jangsaeng Kim, Sung Yun Woo, Jong-Ho Lee

**Affiliations:** 1 Department of Electrical and Computer Engineering and Inter-University Semiconductor Research Center, Seoul National University, Seoul, 08826, Republic of Korea; 2 School of Electronics Engineering, Kyungpook National University, Daegu, 41566 Korea; 3 Department of System Semiconductor engineering, Sogang University, Seoul, 04107 Korea

**Keywords:** V-NAND, V-AND, Area, Energy, Latency

## Abstract

Compute-in-memory (CIM) has emerged as a promising solution to mitigate the data movement bottleneck in von Neumann architectures. While vertical NAND (V-NAND) flash memory has been explored for CIM, its structural constraints, including pass-bias overhead and interconnect parasitic capacitances, limit energy efficiency. In this work, we present a comprehensive comparison between V-NAND and vertical AND (V-AND) flash memory for CIM applications. Analytical modeling and experimental validation demonstrate that V-AND achieves superior energy efficiency, particularly with low-inference-count regimes and increased stack height, by eliminating the bias pass requirement. These results demonstrate that V-AND offers compelling advantages over V-NAND, establishing it as a promising candidate for energy-efficient, scalable, and fast CIM accelerators.

**Graphical abstract:**

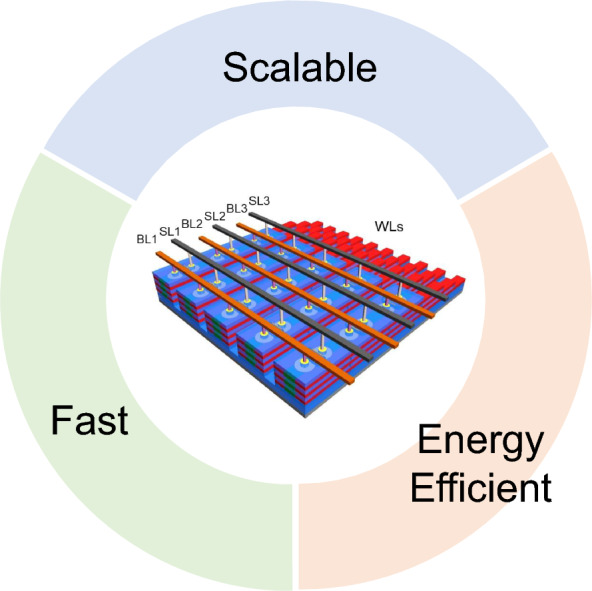

## Introduction

With the advancement of AI, performance surpassing that of humans has emerged across various fields, such as vision and language processing [1–3]. Performing massive AI computations on CPUs/GPUs with a von Neumann architecture is inefficient due to data movement between memory and the digital-based processing units [4]. To solve the problems, there has been growing interest in compute-in-memory (CIM) [5] based on analog computations within nonvolatile memories, such as flash [6], ferroelectric [7], and ReRAM [8]. However, analog computation using a two-dimensional (2d) array suffers from low density in cells and wiring. To address the limitation, recent studies have proposed CIM architectures using commercially mass-produced vertical NAND (V-NAND) flash memory [9, 10]. Since V-NAND flash memory is optimized for conventional memory operations and has difficulty in highly parallel access, we previously developed a rounded-double channel (RDC) vertical AND (V-AND) flash memory to overcome the constraints [11]. There have been various V-AND flash memory structures, including RDC-based and buried diffusion line-based approaches [12, 13]. In conventional RDC-based structure [12], the double channels share an inner gate electrode, making it difficult to independently control the two channels and limiting electrostatic controllability due to the gate being positioned inside the channel. In contrast, the proposed V-AND flash memory array in our previous work [11] separates the gated electrodes for each channel, enabling independent operation. Meanwhile, in buried diffusion line-based approach [13], the channels are not vertically isolated. Therefore, as the number of stacked layers increases, accumulated off-current imposes a fundamental scalability limitation.

Yet, there has been no comprehensive comparison between the V-NAND structure and the V-AND structure from a CIM perspective in terms of density, energy consumption, and latency. Although V-NAND flash cells are in series through multiple floors within a single channel hole, the presence of source-line (SL) trenches and dummy holes requires a precise evaluation of density when comparing with V-AND. Moreover, these structural differences cause variations in the lengths of interconnects such as SL, bit-line (BL), and word-line (WL), leading to different parasitic capacitances. In addition, since V-NAND flash memory requires a pass bias on non-selected WLs while V-AND flash memory does not, it is necessary to analyze the difference in energy consumption. Despite these differences, prior studies [11, 14] have assessed performance only for V-AND in isolation, without analyzing how each metric compares against V-NAND. In this article, for the first time, we compare V-NAND and V-AND flash memory to determine which is superior in terms of density, energy consumption, and latency. This study is expected to provide insights for optimizing CIM operations from various perspectives when extending to 3D nonvolatile memories.

## Background

**Fig. 1:**
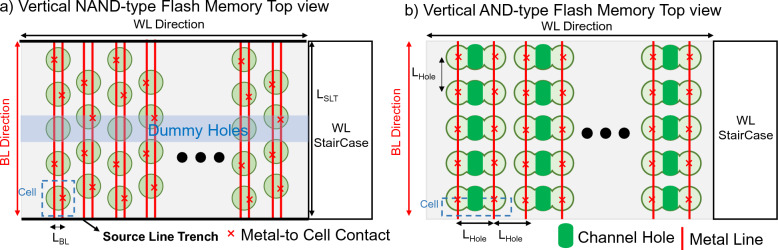
Top views of **a** V-NAND and **b** V-AND flash arrays. V-NAND uses SL trenches and dummy holes for string select, while V-AND employs alternating dummy holes to isolate WLs

To evaluate area and energy consumption, it is important to define the features of the V-NAND and V-AND flash memories fairly. Figures 1a and b show the top views of the V-NAND and V-AND flash memories, respectively. As shown in Fig. 1a, the V-NAND array consists of a defined number of channel holes separated by SL trenches, and the middle row is etched as dummy holes to enable drain select operation [15]. For V-NAND, the BL pitch is known to be formed much tightly by spacer-patterning technology [15]. The SL trench pitch (*L*_SLT_) and BL pitch (*L*_BL_) determine the cell density and interconnect lengths. For the V-AND flash memory array, the top view is shown in Fig. 1b. Dummy holes alternate in each row to separate the WLs, and the hole-to-hole pitch (*L*_Hole_) and diameter are defined identically to those of the V-NAND flash memory array. For the BLs and SLs, the pitch is set equal to *L*_Hole_, because in the V-AND structure, source and drain holes are formed on either side and contacted individually. The defined dimensions are summarized in Tables 1 and 2. In the case of the V-AND, each channel hole contains two cells per layer, and this is considered when comparing densities. Area per cell normalized with the number of floors is calculated as follows:

1 $$ {\text{Area per cell }}\left( {{\mathrm{NAND}}} \right){\text{ }} = L_{{{\mathrm{SLT}}}} \times L_{{{\mathrm{BL}}}} \times {\text{ }}0.{\mathrm{5}} $$

2 $$ {\text{Area per cell }}\left( {{\mathrm{AND}}} \right){\text{ }} = {\text{ 4 }} \times L_{{{\mathrm{Hole}}}} \times L_{{{\mathrm{Hole}}}} \times {\text{ }}0.{\mathrm{5}} $$

**Table 1:** Assumed V-NAND/AND parameters

Parameters	Value
Bit line pitch (*L*_BL_)	40 nm (NAND) /300 nm (AND)
Hole pitch (*L*_Hole_)	150 nm
Staircase pitch (*L*_Stair_)	100 nm
SL trench pitch (*L*_SLT_)	1.4 µm
Floor height (*L*_Height_)	60 nm
Hole diameter (*L*_Dia_)	100 nm
BL read voltage (*V*_ReadBL_)	1 V
WL read voltage (*V*_ReadWL_)	2 V
Pass voltage (*V*_Pass_)	5 V
Line capacitance (*C*_Line_)	0.1 fF/µm
Gate capacitance (*C*_Gate_)	3 fF/ µm^2^
Number of floors (*N*_Floor_)	32/64/96/128
# of BLs / WLs (*N*_BL_ / *N*_WL_)	128/256/512/1024
# of SLTs in NAND (*N*_SLT_)	64/128/256/512
Read time (*T*_read_)	5 ns
Drain capacitance (*C*_Drain_)	1 fF (per 1 µm Width)
# of Select cells (*N*_Select_)	3
On/Off current (*I*_On_/*I*_Off_)	10 nA / 1 pA

**Table 2:** Summary of parameters used in this work

Parameters		Definition
*L*_BL_	Bit-line pitches of V-NAND and V-AND flash memory	
*L*_TotBL_	Total bit-line length of V-NAND and V-AND flash memory	
*L*_TotWL_	Total word-line length of V-NAND and V-AND flash memory	
*L*_Hole_	Center-to-center hole pitch of V-NAND and V-AND flash memory	
*L*_Stair_	Staircase pitch of V-NAND flash memory	
*L*_SLT_	Source-line trench pitch of V-NAND flash memory	
*L*_Height_	Height of each floor in V-NAND and V-AND flash memory	
*L*_Dia_	Hole Diameter of V-NAND and V-AND flash memory	
*A*_Gate_	Gate area of each cell in V-NAND and V-AND flash memory	
*V*_ReadBL_	Bit-Line Read Voltage of V-NAND and V-AND flash memory	
*V*_ReadWL_	Word-Line Read Voltage of V-NAND and V-AND flash memory	
*V*_Pass_	Pass bias of V-NAND flash memory	
*C*_Line_	Parasitic wire capacitance of V-NAND and V-AND flash memory per unit length	
*C*_Gate_	Gate capacitance of V-NAND and V-AND flash memory per unit area	
*C*_*Tot*WL_	Total Gate capacitance of V-NAND and V-AND flash memory	
*C*_*TotB*L_	Total Bit-line capacitance of V-NAND and V-AND flash memory	
*C*_Drain_	Drain capacitance of each cell in V-NAND and V-AND flash memory	
*N*_Floor_	Number of Floors of V-NAND and V-AND flash memory	
*N*_WL_ / *N*_BL_	Top view array dimensions of V-NAND and V-AND flash memory	
*N*_SLT_	Number of source-line trenches in V-NAND flash memory	
*T*_read_	Read time in V-NAND and V-AND flash memory	
*N*_Select_	Number of select cells in V-NAND flash memory	
*N*_Operation_	Number of parallel read operations in V-NAND and V-AND flash memory	
*E*_Set_	Word-line setting energy consumption in V-NAND flash memory	
*E*_WL_	Word-line switching energy consumption in V-NAND and V-AND flash memory	
*E*_BL_	Bit-line switching energy consumption in V-NAND and V-AND flash memory	
*E*_Cell_	Energy consumption by cell current in V-NAND and V-AND flash memory	
*E*_Sense_	Energy consumption by sense-amplifier in V-NAND and V-AND flash memory	
*E*_Tot_	Total Energy consumption in V-NAND and V-AND flash memory	
*I*_Cell_	Cell current flowing to V-NAND and V-AND flash memory cells	
*I*_On_/*I*_Off_	On/off current of V-NAND and V-AND flash memory	

Since each BL supports two cells via the drain select operation, in V-NAND flash memory, multiplying *L*_BL_ by the *L*_SLT_ and then dividing by two defines the cell area. In V-AND flash memory, since dummy holes alternate between rows (× 2), the pitch between BLs (*L*_BL_) is twice the L_Hole_ (× 2), and each channel hole holds two cells per floor (× 0.5), the resulting cell area is larger. A comparison shows that the AND structure has roughly 60% greater cell area than the NAND structure. It is worth noting that commercially fabricated V-NAND architectures still appear to be largely based on the structure reported in [15], without substantial structural deviation. Therefore, the geometric assumptions used in this analysis remain representative of current industrial V-NAND implementations.

**Fig. 2:**
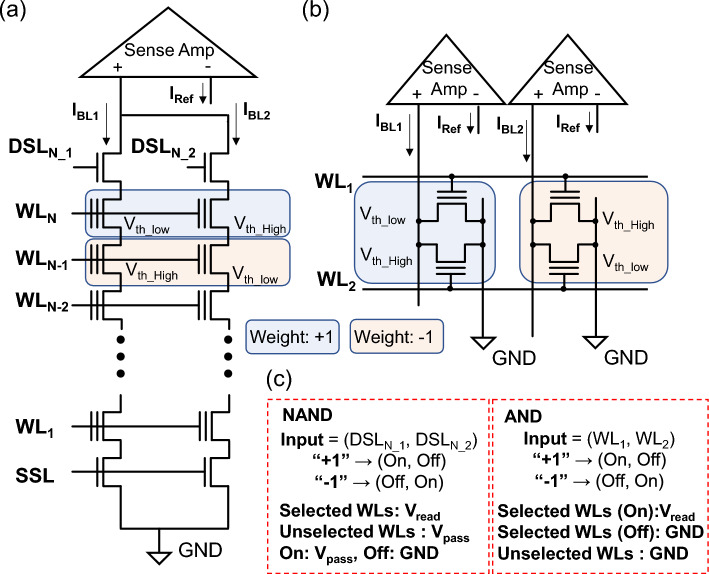
CIM operation schemes using flash cells. **a** V-NAND string structure with drain select lines and sense amplifier readout. **b** V-AND structure where paired WLs encode ± 1 weight. **c** Input encoding and biasing conditions for representing binary weights in NAND and AND arrays

Now, we compare the performance of V-NAND and V-AND flash memories in CIM operations. For a fair evaluation, we assume the same neural network model, a binary neural network (BNN). The BNN scheme follows the approach proposed in prior studies (see Figs. 2a and b): two cells are paired to represent a single weight, storing either a high threshold voltage or a low threshold voltage [16, 17]. An input signal of + 1 or − 1 is applied by driving one of the corresponding two drain select lines (DSLs) or WLs to a high voltage and the other to ground.

Specifically, in V-NAND case, one of the DSLs is set high while the other is grounded [16]. In V-NAND, a BNN can activate only one floor at a time through a WL selected within each DSL pair. All other floors’ WLs are biased in pass voltage. When the DSL is driven to a high voltage and a low-threshold-voltage cell lies in the same string, current flows to the BL, encoding + 1. When the DSL is driven high and a high-threshold-voltage cell lies in the same string, no current reaches the BL, encoding − 1.

In V-AND case, one of the two WLs is set high and the other is grounded [17]. Biasing a low-threshold-voltage cell with a high voltage turns it on and conducts an on-current, encoding + 1. In contrast, when a high-threshold-voltage cell is biased at high voltage while the paired low-threshold voltage cell is held at low voltage, no current flows, encoding − 1. The CIM operating parameters are summarized in Table 1 [18–21]. Under the conditions, we evaluate the energy consumed by V-NAND and V-AND flash memories during BNN operation.

**Fig. 3:**
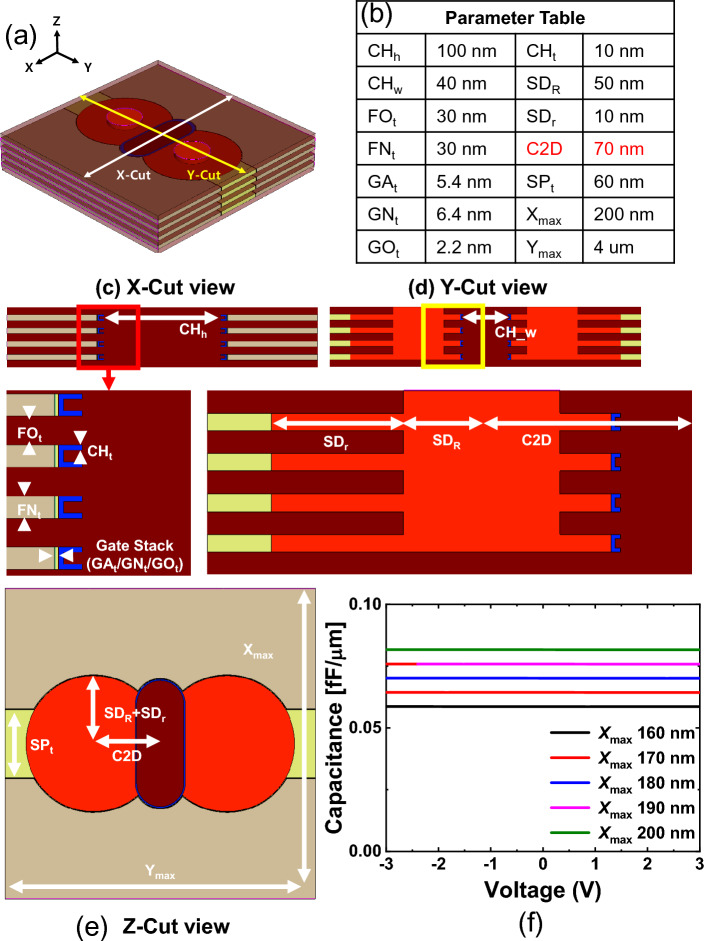
**a** Three-dimensional schematic of V-AND flash memory cell indicating the X- and Y-cut planes. **b** Assumed V-AND flash memory cell feature dimensions. **c** X-cut view, **d** Y-cut view, and **e** Z-cut view of TCAD structure used to evaluate the gate, drain, and wire parasitic capacitances of the V-AND flash cells. **f** Extracted parasitic capacitance of V-AND flash memory

As shown in Fig. 2, various components contribute to the energy consumption during the operation of V-NAND and V-AND flash memories. The energy required to charge WLs, BLs, and DSLs, the energy consumed by the sense amplifier, and the energy consumed by current flow through synaptic devices contribute to the total energy consumption. Gate, drain, and parasitic wire capacitances are evaluated from prior references [16–18] and TCAD simulations, as shown in Fig. 3. Current levels are determined based on measured data and reference [14, 16]. The energy consumption of the sense amplifier is estimated using SPICE simulation results. The SPICE simulation is based on our 350 nm process library (SNU035CMOS). The wire lengths of WLs and BLs are calculated based on the Fig. 1 layout, and a parasitic capacitance of 0.1 fF per micrometer [18], which is also verified by our TCAD simulation (see Fig. 3), is applied. The detailed calculation process of wire length and capacitance elements is as follows:

3 $$ {\text{WL Length }}\left( {L_{{{\mathrm{TotWL}}}} } \right){\text{ }} = L_{{{\mathrm{BL}}}} \times N_{{{\mathrm{BL}}}} $$

4 $$ {\text{BL Length }}\left( {L_{{{\mathrm{TotBL}}}} } \right){\text{ }} = L_{{{\mathrm{SLT}}}} \times N_{{{\mathrm{SLT}}}} \left( {{\mathrm{NAND}}} \right),L_{{{\mathrm{Hole}}}} \times N_{{{\mathrm{WL}}}} \left( {{\mathrm{AND}}} \right) $$

5 $$ C_{{Tot{\mathrm{WL}}}} = A_{{{\mathrm{Gate}}}} \times C_{{{\mathrm{Gate}}}} \times N_{{{\mathrm{BL}}}} + L_{{{\mathrm{TotWL}}}} \times C_{{{\mathrm{Line}}}} $$

6 $$ \begin{aligned} C_{{Tot{\mathrm{BL}}}} = & C_{{{\mathrm{Drain}}}} \times N_{{{\mathrm{WL}}}} + L_{{{\mathrm{TotBL}}}} \times C_{{{\mathrm{Line}}}} \left( {{\mathrm{NAND}}} \right), \\ & C_{{{\mathrm{Drain}}}} \times N_{{{\mathrm{WL}}}} \times N_{{{\mathrm{Floor}}}} + L_{{{\mathrm{TotBL}}}} \times C_{{{\mathrm{Line}}}} ~\left( {{\mathrm{AND}}} \right) \\ \end{aligned} $$

The total capacitance being charged and discharged is computed by summing the parasitic, gate, and drain capacitances connected to each line. *A*_Gate_ refers to the area of the gate of the flash memories. *N*_WL_ and *N*_BL_ denote the vertical and horizontal array dimensions, respectively, in the top view layout of the V-NAND and V-AND flash memory arrays shown in Fig. 1. Therefore, in V-NAND, *N*_WL_ corresponds to twice *N*_SLT_, whereas in V-AND, it denotes the number of WLs visible along the vertical direction in the top view. *C*_TotWL_ and *C*_TotBL_ refer to the total capacitance of the WLs and BLs in V-NAND/AND flash memory array, respectively. *N*_Floor_ refers to the number of floors of V-NAND and V-AND flash memory. In this context, the method for calculating *C*_TotBL_ differs between V-NAND and V-AND. In V-NAND, when the BL parasitic capacitance and the drain capacitances of the connected cells are summed, only the capacitance of the cells directly connected to the BL is included. In contrast, in V-AND, since the drains of the cells in all floors are connected to the BL, the *N*_Floor_ term is multiplied. Energy consumption with the number of multiply-and-accumulate (MAC) cycles in BNN is calculated as follows:

7 $$ E_{{{\mathrm{Set}}}} = C_{{{\mathrm{TotWL}}}} \times {\text{ }}(N_{{{\mathrm{Floor}}}} + N_{{{\mathrm{select}}}} {\text{ - 1}}){\text{ }} \times N_{{{\mathrm{WL}}}} \times {\text{ }}\left( {V_{{{\mathrm{pass}}}} } \right)^{{\mathrm{2}}} \left( {{\mathrm{NAND}}} \right) $$

8 $$ E_{{{\mathrm{WL}}}} = C_{{{\mathrm{TotWL}}}} \times N_{{{\mathrm{WL}}}} \times {\text{ }}\left( {V_{{{\mathrm{Pass}}}} - V_{{{\mathrm{ReadWL}}}} } \right)^{{\mathrm{2}}} $$

9 $$ E_{{{\mathrm{BL}}}} = C_{{{\mathrm{TotBL}}}} \times N_{{{\mathrm{BL}}}} \times {\text{ }}\left( {V_{{{\mathrm{ReadBL}}}} } \right)^{{\mathrm{2}}} $$

10 $$ E_{{{\mathrm{Cell}}}} = \sum I_{{{\mathrm{Cell}}}} \times T_{{{\mathrm{Read}}}} \times V_{{{\mathrm{ReadBL}}}} $$

11 $$ {\mathrm{E}}_{{{\mathrm{sense}}}} = {\text{ 325 fJ }} \times N_{{{\mathrm{BL}}}} $$

12 $$ \begin{aligned} E_{{{\mathrm{Tot}}}} = & {\text{ }}\left( {E_{{{\mathrm{cell}}}} + E_{{{\mathrm{sense}}}} + E_{{{\mathrm{Ref}}}} + E_{{{\mathrm{BL}}}} + E_{{{\mathrm{WL}}}} } \right){\text{ }} \\ & \times N_{{{\mathrm{Operation}}}} + E_{{{\mathrm{Set}}}} \left( {{\mathrm{NAND}}} \right), \\ & \left( {E_{{{\mathrm{cell}}}} + E_{{{\mathrm{sense}}}} + E_{{{\mathrm{Ref}}}} + E_{{{\mathrm{BL}}}} + E_{{{\mathrm{WL}}}} } \right){\text{ }} \\ & \times N_{{{\mathrm{Operation}}}} \left( {{\mathrm{AND}}} \right) \\ \end{aligned} $$

**Fig. 4:**
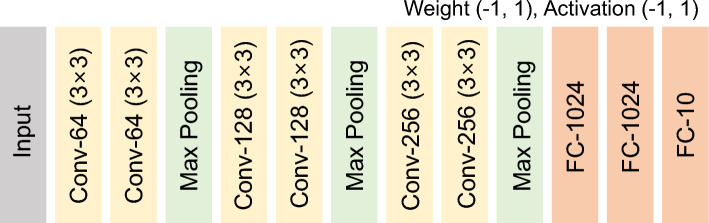
Binary neural network (BNN) architecture.VGG-9 BNN with three convolutional blocks (two 3 × 3 convolutional layers + 2 × 2 max-pooling), followed by three fully connected layers

*E*_set_ denotes the energy required in V-NAND to place non-selected WLs at the pass bias. It is obtained by evaluating the dynamic energy of the WLs. Since *E*_Set_ requires precharging all unselected WLs as well as the WL corresponding to the selected cell, the term (*N*_Floor_ + *N*_select_−1) should be included. *N*_select_ denotes the number of select cells contained in each V-NAND flash memory string. Furthermore, because *C*_TotWL_ and *C*_TotBL_ do not themselves include the array dimensions, they should both be multiplied by *N*_WL_ and *N*_BL_, respectively, which represent the array dimensions in the top view layout. *V*_pass_ represents the pass bias voltage applied to unselected WLs and select lines during read or CIM operation. Because V-AND does not require pass biasing, *E*_set_ is zero for V-AND. *E*_WL_ is the per-MAC energy due to charging and discharging the WL bias applied to the selected layer. *V*_ReadWL_ denotes the voltage applied to the selected WL during read operation. *E*_BL_ represents the dynamic energy consumed at the BL. *C*_BL_ denotes the capacitance of the BL. *E*_sense_ is the total energy consumed by the current sense amplifiers used in V-NAND and V-AND flash memory. It is obtained by multiplying the single operation sensing energy, 325 fJ, by the total number of BLs, *N*_BL_. *E*_Tot_ represents the total energy consumption and is obtained by summing all the energy components described above. *E*_Ref_ denotes the energy consumed by the reference current during BNN operation. It is calculated as the product of the reference current magnitude, the supply voltage, and the operation time. *E*_cell_ is the static energy dissipated in the V-NAND/AND flash memory cells. *E*_cell_ is calculated as the product of the supply voltage, the measured current, and the duration over which the *I*_cell_ (cell current) flows. The current values are extracted from the inference operation of the BNN structure in Fig. 4. *N*_operation_ is the number of MAC cycles (one MAC cycle executes the computation for a single layer as illustrated in Fig. 2). The total energy is then obtained by multiplying the terms that scale with *N*_operation_ and adding any terms that do not.

## Results and discussion

**Fig. 5:**
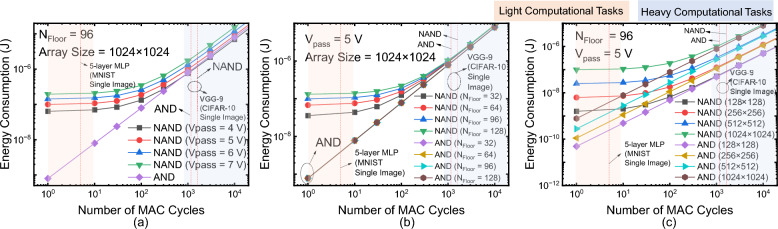
Energy consumption comparison of V-NAND and V-AND CIM operations. **a** Effect of pass voltage on NAND energy. **b** Dependence on stack height. **c** Dependence on array size, showing V-AND maintains higher efficiency across conditions

V-NAND and V-AND flash memories exhibit fundamental differences in their operation schemes. In V-NAND flash memory, while applying read bias to the selected cell, pass bias is also applied to all unselected cells in the same strings. It results in large energy consumption during the WL precharge [21]. Therefore, it is energy-efficient when multiple read operations are performed after a single precharge. In contrast, V-AND flash memory does not require pass bias on unselected cells, eliminating this energy overhead. It leads to a substantial energy disadvantage for V-NAND in cases where only a limited number of read operations are performed after each precharge. As shown in Fig. 5a, even when using a relatively low pass bias of 4 V, V-NAND exhibits significantly lower energy efficiency compared to V-AND when the number of read operations is small. This inefficiency becomes more significant as the pass bias increases. Furthermore, as shown in Fig. 5b, the impact of this energy overhead increases with the number of floors. Since more pass-biased cells are involved as the stack height increases, the energy consumed grows correspondingly. In contrast, V-AND flash memory is much less sensitive to stack height because it does not require pass bias, and the resulting increase in energy is mainly associated with a moderate rise in the drain-capacitance component of the total bit-line capacitance. This trend remains consistent regardless of array size, as shown in Fig. 5c. Across a range of array dimensions, from 128 × 128 to 1024 × 1024, V-NAND consistently demonstrates lower energy efficiency than V-AND in low-read regions.

**Fig. 6:**
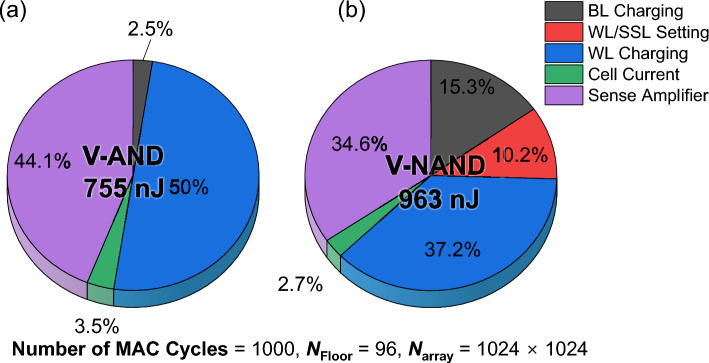
Energy breakdown for MAC operations in V-AND and V-NAND. Proportion of total energy spent on cell current, BL/WL charging, sense/reference, and WL/SSL setting. V-AND has no WL/SSL charging term, while V-NAND shows a non-negligible share from WL/SSL charging

Figure 6 compares the energy breakdown of V-NAND and V-AND for 1,000 MAC cycles, assuming a pass bias voltage of 5 V, 96 floors, and a 1024 × 1024 array dimension. The total energy is 755 nJ for V-AND and 963 nJ for V-NAND. Thus, V-NAND consumes 27% more energy than V-AND. In V-AND, the WL dynamic energy consumption component is larger, whereas in V-NAND the share of WL dynamic energy consumption is smaller. This follows from the architecture in Fig. 1. V-AND inherently has longer *L*_TotWL_, which leads to larger WL capacitance, while V-NAND has longer BLs because repeated SL trenches increase *L*_BL_. Therefore, dynamic energy consumption by BLs dominates more in V-NAND. V-AND has no WL and SSL setting energy, which accounts for about 10% of the total energy consumption of V-NAND. If more MAC cycles are executed per single WL/SSL setting, that fraction will proportionally decrease. Structurally, current in V-AND flows through metal SL and BL lines, whereas in V-NAND it must pass through a series string of unselected cells. Assuming fabrication under identical process conditions, this implies that V-AND can potentially achieve the same cell current with lower *V*_BL_ or lower *V*_ReadWL_ due to reduced series resistance. As a result, the energy consumption part associated with cell current could be further reduced in V-AND. However, since the cell current-related energy accounts for only a minor portion of the total energy in Fig. 6, such bias optimization would not dramatically alter the overall energy trend, although it would further favor V-AND.

In both cases, the sense amplifier energy is non-negligible. In this study, we assume a current sense amplifier because prior BNN related works commonly adopt current sensing schemes [14, 16]. If a voltage sense amplifier were assumed instead, the absolute sensing energy differ depending on the specific circuit implementation and sensing margin. Both current- and voltage-sensing schemes have demonstrated competitive energy efficiency in modern designs, and their relative advantage is largely implementation-dependent rather than fundamentally determined by the sensing principle itself. Nevertheless, voltage sensing determines the output by precharging the BL and then measuring the discharge behavior, which is governed by RC time constants [22]. As a result, depending on the detailed sensing circuit implementation, voltage sensing can exhibit slower operation than current sensing. Therefore, while voltage sensing may in some cases offer improved energy efficiency, the potential speed trade off should be considered in the design.

**Fig. 7:**
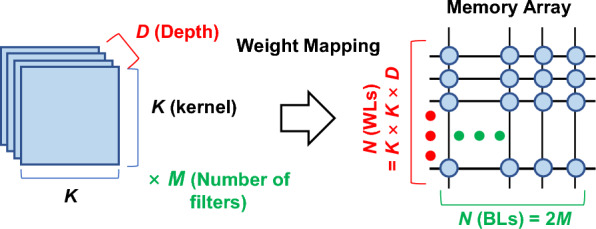
Mapping a *K* × *K* × *D* convolution with *M* filters onto an *N* × *N* memory array

Furthermore, as neural networks grow, each layer often exceeds the size of a single compute array. In V-NAND–based designs, such oversized neural layers must be processed sequentially across multiple arrays, which increases the number of accesses per layer. For convolutional layers, we adopt the weight-mapping scheme in Fig. 7 [23]. With *N* × *N* arrays, a convolutional layer with kernel size *K*, input feature depth *D*, and output channel depth *M* requires ceil(*K* × *K* × *D*/*N*) × ceil(2* M*/*N*) arrays [23]. The factor of 2 on *M* arises because a BNN uses two cells per weight. If *K* × *K* × *D* or 2* M* exceeds *N* in an *N* × *N* string array V-NAND flash, the layer cannot be executed within a single MAC cycle and must be split over multiple sequential cycles. This indicates that V-NAND implementation requires ceil(*K* × *K* × *D*/*N*) × ceil(2* M*/*N*) × *Stride* cycles for the single-layer processing. Due to the stride operation in the convolution layer, the same MAC cycles on the same memory array must be repeated multiple times. The stride term is determined by the number of pixels delivered to the layer and by how many pixels can be processed simultaneously in a single MAC cycle [23]. This can be written as follows:

13 $$ Floors\left( \# \right){\text{ }} = {\text{ ceil}}\left( {K \times K \times D/N} \right){\text{ }} \times {\text{ ceil}}\left( {{\mathrm{2}}M/N} \right) $$

14 $$ Stride = pixel\left( \# \right){\text{ }}/{\text{ min}}\left( {{\mathrm{ceil}}\left( {N/\left( {K \times K \times D} \right)} \right){\text{ }} \times {\text{ ceil}}\left( {N/{\mathrm{2}}M} \right)} \right) $$

**Fig. 8:**
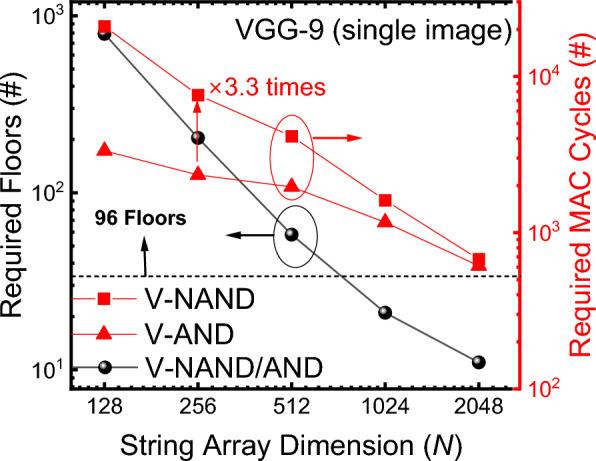
Required floors to store all weights (left axis) and required MAC cycles per inference (right axis) versus array size *N*. Larger *N* reduces both the floor count and the cycle count. V-NAND demands more cycles at small *N* due to sequential floor access

For fully-connected layers with input and output dimensions *I* and *O*, a single array suffices when *N* is greater than both *I* and 2*O* (the factor of 2 again reflects two cells per BNN weight). Otherwise, the design requires ceil(*I*/*N*) × ceil(2*O*/*N*) arrays, and, because V-NAND flash memory can activate only one floor at a time, the number of access cycles grows accordingly. For instance, with a 1024 × 1024 array and a fully-connected layer (*I* = 1024, *O* = 1024), two arrays and two access cycles are needed. In contrast, V-AND flash memory supports simultaneous, layer-wise access, completing the same operation in a single access. This is possible because a layer may be distributed across different floors while still permitting parallel access to those floors. This advantage becomes more suitable for larger, higher-performance models, making V-AND flash memory preferable from a latency perspective. Figure 8 shows, as a function of *N*, the number of floors required in V-NAND/AND flash memory to store the parameters and the number of MAC cycles needed for a single BNN inference. As *N* increases, more weight parameters are mapped within a single floor, so the required floor count decreases. For *N* more than 512, all weight parameters of the BNN in Fig. 4 can be stored within 96 floors. For smaller *N*, multiple *N* × *N*, 96-floor V-NAND/AND flash memory arrays would be required. The figure also plots the MAC cycles per BNN inference. Larger *N* allows more computing capability per cycle, reducing the required cycle count. When *N* is large, V-NAND, as V-AND, can store most layers within a single floor, yielding similar MAC cycle counts. When *N* is small, however, V-NAND flash memory often must distribute a single layer across multiple floors and access them sequentially, so its required MAC cycles increase relative to V-AND flash memory.

In summary, assuming the same height, V-AND flash memory exhibits lower density than V-NAND flash memory. Nevertheless, for CIM operation, when the pass bias voltage exceeds 4 V, V-AND flash memory consumes less energy per MAC cycle and is therefore more energy-efficient. In V-NAND operation, the pass bias applied to unselected cells must be sufficiently high to ensure adequate channel formation along the string, thereby maintaining sufficient bit-line current and read margin. As reported in [16], the bit-line current increases with pass bias, indicating that insufficient pass bias can degrade sensing margin and impact inference reliability. Under practical operating conditions where reliable string conduction is required, the pass bias cannot be arbitrarily reduced. In addition, unlike V-AND, the high pass bias required in V-NAND typically necessitates the use of an on-chip charge pump circuit. In previous studies, the energy consumption of the charge pump has been assumed to be 250 nJ per operation, which is non-negligible compared to the total energy consumption [19]. When this overhead is included, the total energy consumption shown in Fig. 6 changes from 755 nJ (V-AND) and 1213 nJ (V-NAND), further widening the energy gap between the two architectures. This operational constraint further strengthens the energy-efficiency advantage of V-AND flash memory in CIM applications. Moreover, V-NAND flash memory suffers substantial WL-setting overhead. When the number of MAC cycles is small, this inefficiency becomes pronounced, making V-NAND flash memory disadvantageous for edge scenarios that perform only a few inferences. From a latency perspective, V-NAND flash memory also requires more MAC cycles because layers must be accessed floor-by-floor sequentially, whereas V-AND flash memory supports simultaneous, layer-wise access. Increasing *N* can narrow the gap by mapping more weights per floor, but this approach demands careful consideration of nonidealities such as IR-drop as *N* grows [14].

## Conclusion

A comparative analysis of V-NAND and V-AND flash memories for CIM applications was presented for the first time. While the V-NAND flash memory has remarkable cell density, the mandatory pass-biasing of unselected cells induces substantial energy overhead. By eliminating the requirement of pass-bias, V-AND flash memory achieves significantly improved energy efficiency. Moreover, because pass-bias is not required, energy efficiency does not degrade as the number of floors increases, which is advantageous for scalability. In addition, unlike the V-NAND architecture, where parallel execution is limited to only a subset of layers, V-AND architecture enables parallel execution across multiple layers, yielding higher parallelism. These findings position V-AND flash memory as a strong alternative to V-NAND flash memory for next-generation CIM, offering a favorable trade-off between density, energy, and latency.

## Data Availability

All relevant data are available within the article. The data generated in this study are provided in Source Data files. All other data are available from the corresponding author upon request.
